# Application of Three-dimensional Visualization Fused with Ultrasound for Percutaneous Renal Puncture

**DOI:** 10.1038/s41598-021-87972-8

**Published:** 2021-04-19

**Authors:** Zhibin Xu, Zhenchi Li, MaoMao Guo, Hao Bian, Tianli Niu, Jiangping Wang

**Affiliations:** 1 Department of Urology, Taizhou People’s Hospital, 366 Taihu Road, Taizhou, 225300 Jiangsu China; 2 Graduate School of Dalian Medical University, No.9 West Section, Lushun South Road, Dalian, 116044 China

**Keywords:** Health care, Medical research, Urology

## Abstract

We present here the three-dimensional (3D) visualization fused with ultrasound and to evaluate its clinical application effect preliminarily. One hundred and eighteen patients with renal calculi in our hospital from September 2017 to December 2019 were prospectively randomized into two groups. The experimental group was treated with percutaneous renal puncture guided by the 3D visualization fused with ultrasound. The control group was treated with percutaneous renal puncture guided by B-ultrasonography (B-US). The puncture time in the experimental versus control group was 4.36 ± 1.28 min versus 10.72 ± 2.94 min (*P* = 0.000), operation time was 65.85 ± 10.63 min versus 81.34 ± 12.52 min (*P* = 0.000), and the loss of hemoglobin was 8.55 ± 3.76 g/L min versus 13.33 ± 5.81 g/L(P = 0.000), and the success rate of establishing the channel at one time was 98.41% versus 81.82% (*P* = 0.002), and the coincidence rate between the channel and the longitudinal axis of the target renal calyx was 88.89% versus 60.00% (*P* = 0.000). The 3D visualization fused with ultrasound could guide precise puncture to target calyces, reduce operation time, bleeding, and difficulty of puncture.

## Introduction

The most important technique during percutaneous nephrolithotomy is precise percutaneous renal puncture^1^. The ideal route for this intervention entails passing through the axis of the targeted renal calyx, which minimizes vascular injury around the calyx and obtains the largest renal endoscopy angle^2,3^. At present, the main techniques for guiding percutaneous renal puncture are plain radiography and B-ultrasonography (B-US)^4,5^. Because radiography and B-US provide only two-dimensional (2D) images, it is impossible to guide the puncture through the axis of the targeted renal calyx that more-precise three-dimensional (3D) imaging could provide, leading to a less than accurate percutaneous renal puncture and an increased risk of bleeding^6^. Medical 3D visualization technology can process 2D computed tomography (CT) images using a computer, converting the 2D images into intuitive, perspective 3D images to display the 3D shapes of human organs and tissues. One of the important functions of 3D visualization is that it can be marked and segmented on digital 3D images, which could be used for navigation during surgery. Therefore, we hypothesized that 3D visualization fused with ultrasound can achieve precise guidance for renal puncture through the axis of the targeted renal calyx.

## Methods

### Patients

After being assessed for eligibility, the patients were given informed consent. 118 patients with renal calculi > 3 cm in volume were selected during September 2017 to December 2019 to participate in this study. All patients met the criteria for undergoing percutaneous nephrolithotomy. All patients were randomly divided into two groups with coin toss: experimental group (n = 63) and control group (n = 55). The 3D visualization fused with ultrasound was used in the experimental group to guide percutaneous renal puncture and only B-US was used in the control group. The assessed parameter included age, BMI, gender, stone volume, and hydronephrosis (Table 1). All methods were carried out in accordance with relevant guidelines and regulations. This study protocol was approved by the Research Ethics Committee of Jiangsu Taizhou People’s Hospital.

**Table 1:** The detailed information of patients.

	Control group (n = 55)	Experimental group (n = 63)	*P* value
Age (year)	41.2 ± 6.8	42.5 ± 6.6	0.353
BMI (kg/m^2^)	23.4 ± 2.7	23.1 ± 3.0	0.728
**Gender**			
Male (n/%)	36/65.5	40/63.5	0.824
Female (n/%)	19/34.5	23/36.5	0.824
Stone volume (cm^3^)	6.68 ± 4.33	7.21 ± 4.62	0.775
**Hydronephrosis**			
No (n/%)	21/38.2	24/38.1	0.992
Mild (n/%)	19/34.5	22/34.9	0.966
Moderate (n/%)	15/27.3	17/27.0	0.972

BMI, body mass index.

### CT scan

**Figure 1:**
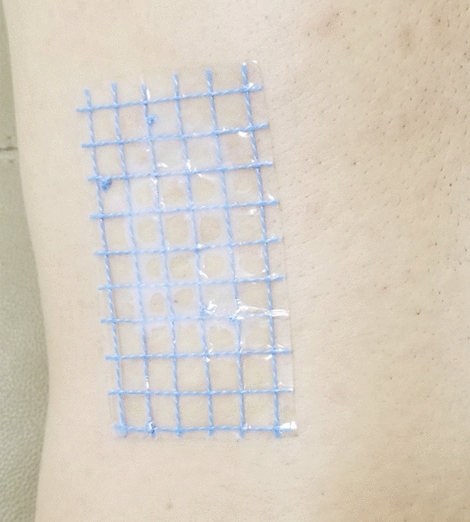
Grid patch placed on the body surface. *A*, *B*, *C*, Front, side, and back views, respectively, of the three-dimensional (3D) visual model of the renal collecting system (red), kidney calculi (green), and grid patch (gold).

In the experimental group, a homemade, grid-shaped body surface patch (10.0 × 6.0 cm), with each square 1.0 × 1.0 cm, was place on, and adhered to, the common area for percutaneous renal puncture (Fig. 1)—i.e., in the area of the inferior rib om the affected side and posterior axillary line before CT scan. All patients in the two groups underwent CT scanning in a standard lateral position at the end of inspiration. Spiral, 256-slice CT was used to scan the upper and middle abdomen, with a slice thickness of 0.5 mm. Structural images were recorded in plain scan phase, arterial phase, venous phase, and excretory phase.

### 3D visualization fused with ultrasound

#### Preoperation

**Figure 2:**
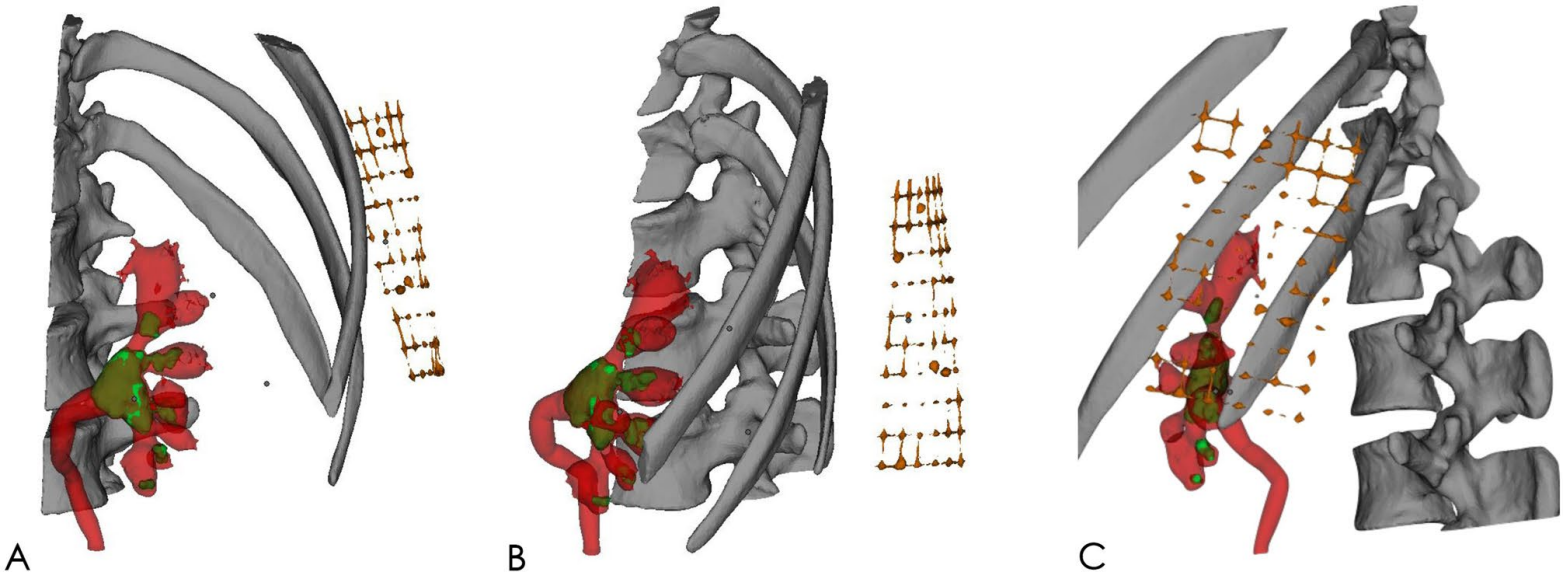
3D visual model of the renal collecting system, renal calculi, and grid patch. (**A**–**C**), Front, side, and back views of the 3D visual model of the renal collecting system (red), kidney calculi (green), and grid patch (gold). The figure was created with Mimics 19.0 (https://www.materialise.com).

The 2D grayscale digital images obtained from CT scan were input into Mimics software (Materialise Corp., Leuven, Belgium) for 3D reconstruction. Image segmentation was carried out using threshold segmentation, the region growth method, and Boolean function and was modified by a multi-layered manual segmentation method. Renal calculi, spine, ribs, and the gridded body surface patch were segmented and reconstructed according to the plain scanning images. The renal contour was then segmented and reconstructed according to the arterial phase images. The renal pelvis secretory phase images were used to segment and reconstruct the collection system. After registration, the model was de-noised, smoothed, and carefully filled. Finally, the 3D visual model of the spinal column, partial rib, grid-shaped body surface patch, renal collecting system, and renal calculi were reconstructed (Fig. 2).

**Figure 3:**
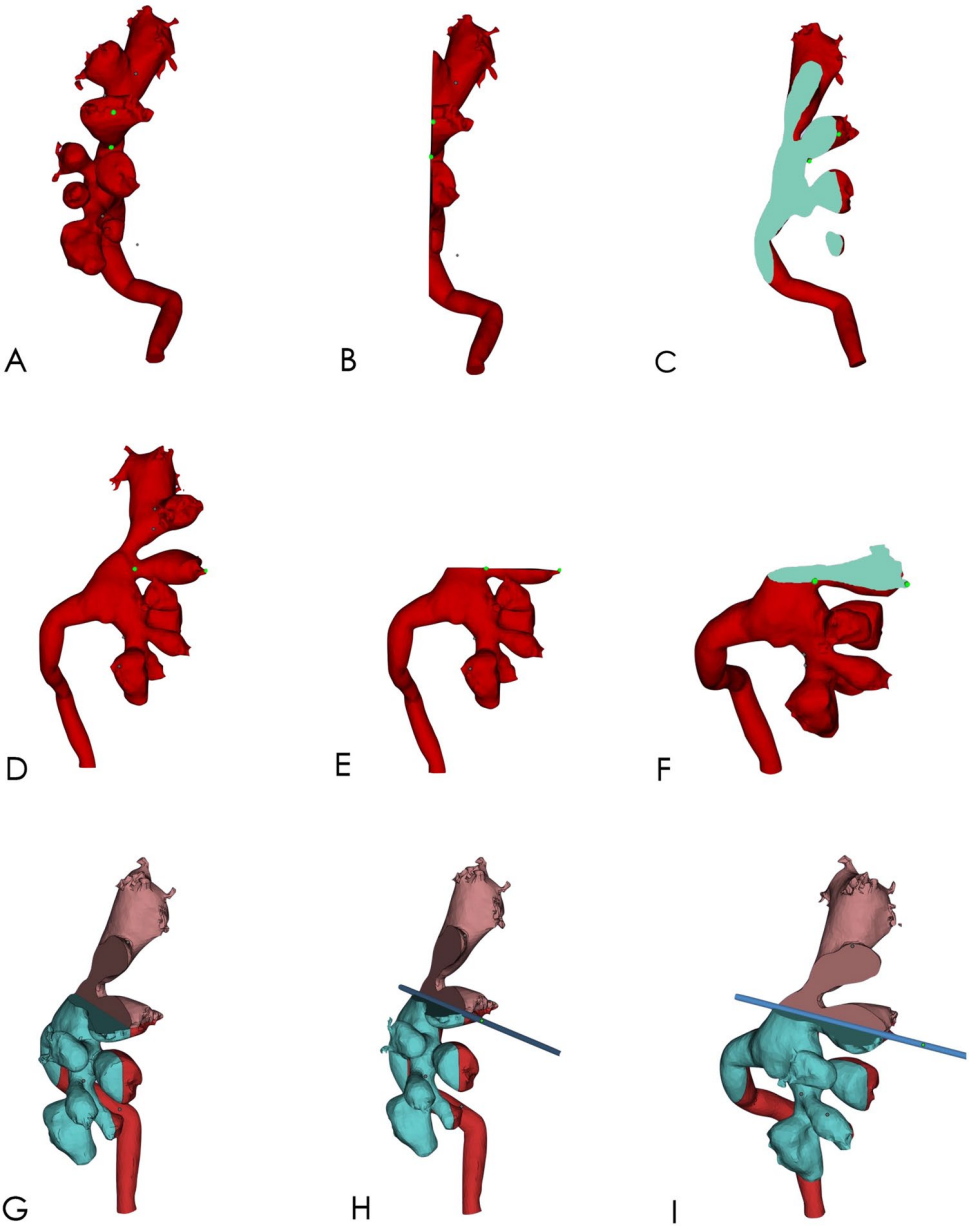
Axis of the targeted renal calyx on the 3D visual model. (**A**–**C**), Obtaining the axial section A of the targeted renal calyx. (**D**–**F**) Obtaining the axial section B of the targeted renal calyx. (**G**–**I**) Cross line of axial sections A and B of the targeted renal calyx is its axis (blue, rod-like). The figure was edited with Magics 20.03 (https://www.materialise.com).

**Figure 4:**
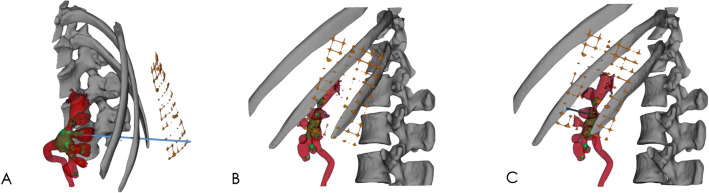
Simulated location of skin puncture points. (**A**–**C**) From different points of view, the axis of the targeted renal calyx (blue, rod-like) extended to the body surface. The point that intersected with the grid surface patch was the point of skin puncture. The figure was edited with Magics 20.03 (https://www.materialise.com).

**Figure 5:**
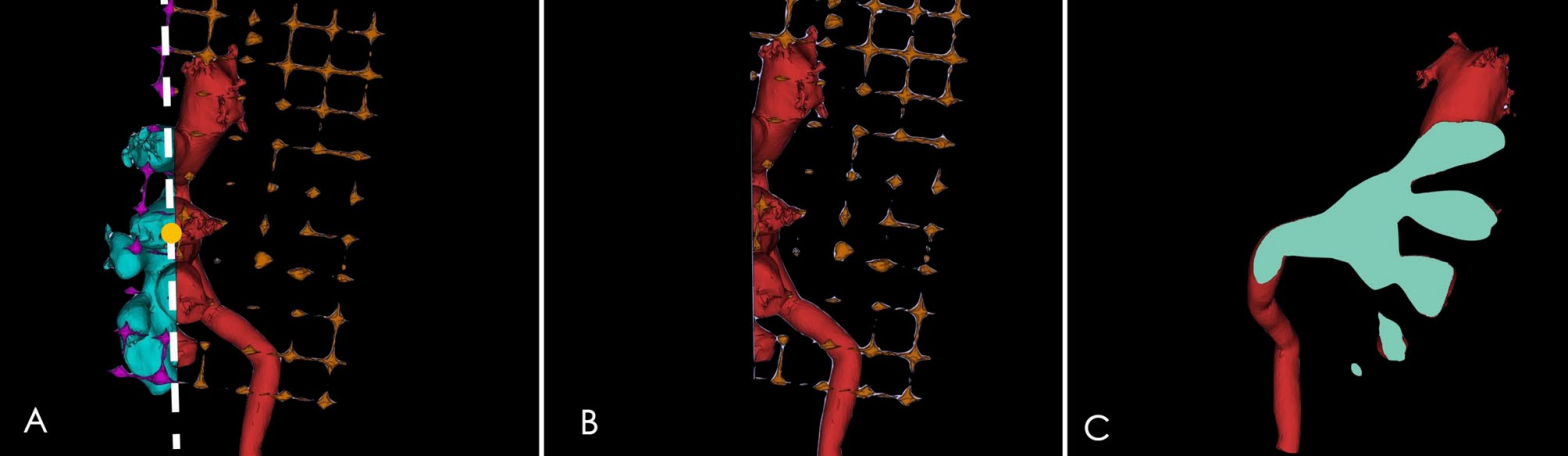
Segmentation of the optimal simulated ultrasonography-guided section of the targeted renal calyx. (**A**,**B**) The section was segmented through the axis of the targeted renal calyx and as close as possible to the axis of the adjacent posterior renal calyx (white line segment). (**C**) Segmented section C (cyan, outlined) shows the targeted renal calyx, renal pelvis, and surrounding renal calyces. The figure was edited with Magics 20.03 (https://www.materialise.com).

**Figure 6:**
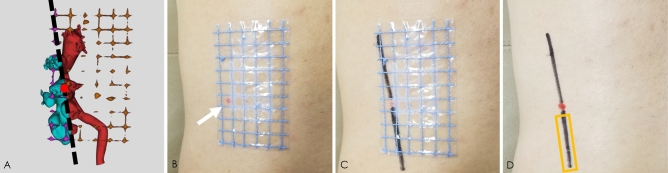
Location of the skin puncture point and ultrasonic probe. (**A**) Skin puncture point (red dot) and tangent of section C are located on the grid patch (black line segment). (**B**) Point of skin puncture (red spot indicated by the white arrow) was located on the skin of the patient using the grid surface patch. (**C**) Tangent of section C, which was restored on the skin of the patient using a grid surface patch (black line segment). (**D**) Tangent (black segment in the golden box) of section C on the grid surface patch, which is where the ultrasonic probe was placed.

After the 3D model was reconstructed with Mimics software, the 3D model was moved to Magics software (Materialise Corp., Leuven, Belgium) for marking and segmentation. First, using the segmentation tool of Magics software, the line between the vertex of the fornix and the midpoint of the calyx was drawn from any side of the model of the targeted renal calyx, and section A (perpendicular to the side of the renal calyx) was drawn along that line (Fig. 3A–C). Another section (B) was obtained from the other side of the targeted renal calyx using the same method (Fig. 3D–F), and the line crossing the two sections was the axis of the targeted renal calyx (Fig. 3G–I). Extending the axis, the point of the line passing through the localization patch was the point at which the skin should be punctured (Fig. 4). Second, we identified the posterior targeted calyx and the adjacent posterior calyces on the 3D visual model of the renal collection system. The axial section C, segregated along the axis of the targeted renal calyx and as close as possible to the adjacent posterior calyx, was the optimal simulated US-guided section of the targeted calyx that could maximally show the relation between the targeted calyx and the targeted pelvis and their surrounding calyces (Fig. 5). Section C crossed the grid to form a tangent that passed through the puncture point. According to the tangent and the position of the puncture point on the grid, the line and puncture point could be accurately restored on the patient’s skin (Fig. 6A–C). The guiding lines on both sides of the puncture point are the ideal position of the ultrasonic probe during the puncture (Fig. 6D).

The above work was completed by two surgeons of our team at one day before the operation. It took about 30 min for each patient. After 5 patients, a general surgeon can be familiar with the 3D software.

#### Intraoperation

**Figure 7:**
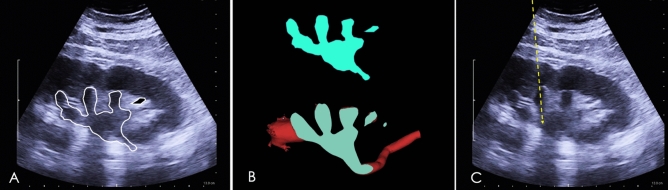
Accurate navigation of the puncture through the axis of the targeted renal calyx. (**A**) Ultrasonographic (US) observation of the section profile (white contour) of the renal collecting system. (**B**) US profile of the renal collecting system (superior blue contour) was highly consistent with the optimal axial section C (inferior blue contour). (**C**) Direction of the ultrasonic probe was now fixed, and the puncture route (yellow arrow) through the center of the targeted renal calyx section was the axis of the targeted renal calyx.

During the operation, in the lateral position on the healthy side, the head of the B-US probe was pressed on the puncture point, swinging the probe after placing it along the location line. The probe was fixed when the 2D US image of the renal pelvis and renal calyx was the same or similar to the image of section C on the computer screen (Fig. 7A,B). The needle was then inserted from the puncture point of the head of the B-US probe. The puncture line of the needle that passed through the central line of the targeted calyx in the 2D US image was the axis of the targeted renal calyx (Fig. 7C). Each patient underwent the surgery in a standard lateral position, and each was punctured at the end of inspiration to avoid complications due to kidney displacement.

#### Postoperation

**Figure 8:**
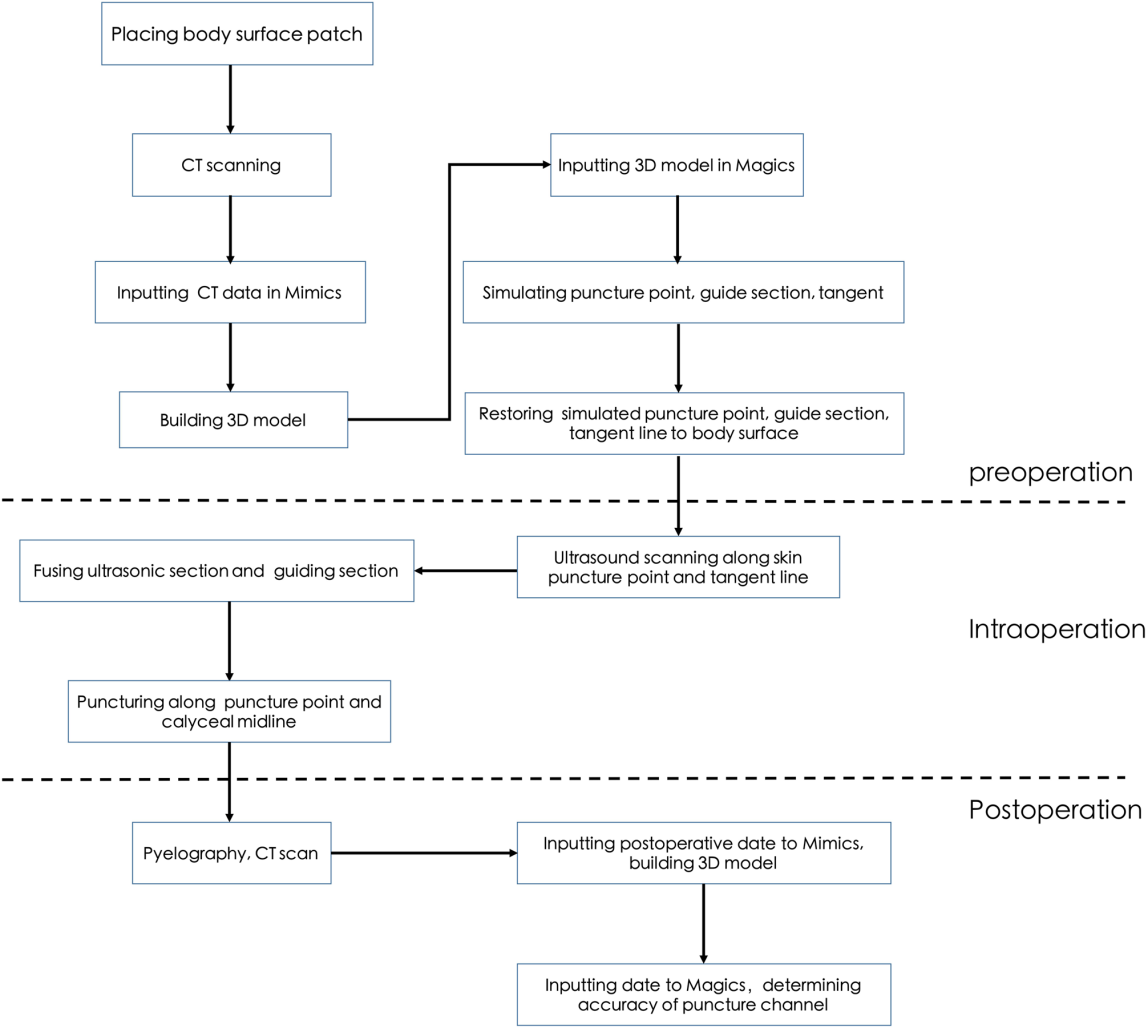
The technical flow chart of 3D visualization fused with ultrasound.

The puncture time was recorded, and the changes in hemoglobin levels before and after the operation were compared. On the third to five postoperative day, contrast agent was injected into the F16 percutaneous renal drainage tube, and the patient underwent CT scan in the lateral position, and the 3D structures of the renal pelvis, renal calyx, and drainage tube were reconstructed. The nephrostomy tube was removed 4–6 days after operation. The drainage tube represented the puncture route. Whether the puncture route passed through the axis of the targeted renal calyx was observed. The puncture passage passes through the target renal calyceal nipple and the skin puncture point was judged to be consistent with the target renal calyx longitudinal axis in the 12 mm around the intersection of the renal calyx longitudinal axis and the skin. The whole technical flow chart of 3D visualization fused with ultrasound was shown in Fig. 8.

### Conventional puncture technique

CT images were read routinely and the target calyces with puncture route were designed in mind with conventional 2D method at one day before operation. During the operation, only B-US was used to locate the puncture point and guide the puncture. In order to drain the renal pelvis, all the patients in the two groups were placed ureteral stents. The other intraoperative and postoperative steps were same as those in the experimental group.

### Observation indicators

The operation time, puncture time, hemoglobin loss, the success rate of one-time establishment of the channel, the coincidence rate between the channel and the longitudinal axis of the target renal calyx were compared between the two groups, and the stone clearance rate, blood transfusion rate and interventional embolization hemostasis rate were observed. Blood samples were collected at admission and 48 h after operation. The decrease of hemoglobin was calculated as hemoglobin loss. No residual stones or meaningless residual stones less than 4 mm in CT images were regarded as stone clearance.

### Statistical methods

The primary end point is the coincidence rate of puncture passage. Based on power of 90%, α = 0.05, the least sample size needed in each group is 48. SPSS 17 (IBM Corp., Armonk, NY, USA) statistical software was used for data processing, measurement data were expressed by $\overline{x}$ ± S, *t*-test was used for comparison between groups, percentage was used for counting data, ${X}^{2}$ test was used for comparison between groups. *P* < 0.05 indicates that the difference is statistically significant.

## Results

**Figure 9:**
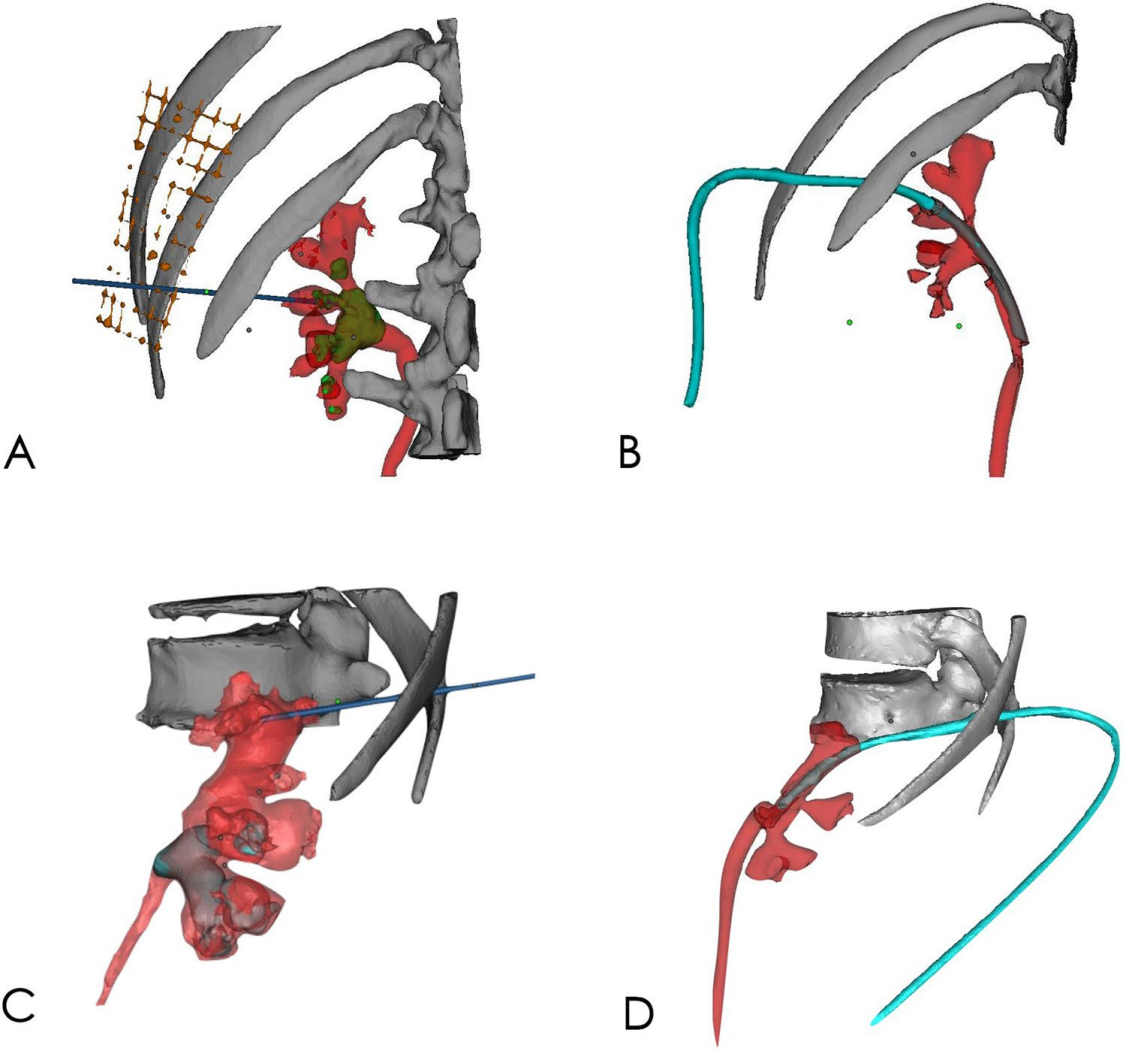
3D visual model verifies that the puncture route passes precisely through the axis of the targeted renal calyx. (**A**,**C**) Preoperative 3D visual model of the renal collecting system, renal calculi, and the axial of the targeted renal calyx. (**B**,**D**) Postoperative 3D visual model shows that the puncture route passed precisely through the axis of the targeted renal calyx, which was highly consistent with that of the simulated axis of the targeted renal calyx before the operation. The figure was created with Mimics 19.0 (https://www.materialise.com).

There was no significant difference in age, gender, BMI, stone volume and degree of hydronephrosis between the two groups (Table 1). Under the guidance of the 3D visualization, the targeted renal calyces of the patients were punctured quickly and successfully in accordance with the puncture points and routes located before operation. Intraoperative observation showed that the puncture route entered the targeted renal calyx from the center of the papilla of the targeted renal calyx, and the puncture route faced the renal calyx opening directly. Postoperative 3D reconstruction showed that the puncture channels passed through the axis of the targeted renal calyx (Fig. 9).

The puncture time, operation time and the loss of hemoglobin in the experimental group were lower than those in the control group, and the success rate of establishing the channel at one time and the coincidence rate between the channel and the longitudinal axis of the target renal calyx were higher than those in the control group, and the difference was statistically significant (Table 2). There was no difference in the stone clearance rate, the blood transfusion rate and the interventional embolization rate between the experimental group and the control group in statistics (Table 3), but the powers were 17%, 6%, and 6% respectively evaluated by the further statistics.

**Table 2:** Comparison of operation between the two groups.

Group	Number	Operation time (min)	Puncture time (min)	Hemoglobin loss (g/L)	Primary channel success rate (number/%)	Coincidence rate of puncture passage (number/%)
Experimental group	63	65.85 ± 10.63	4.36 ± 1.28	8.55 ± 3.76	62/98.41	56/88.89
Control group	55	81.34 ± 12.52	10.72 ± 2.94	13.33 ± 5.81	45/81.82	33/60.00
*P value*	0.000	0.000	0.000	0.002	0.000	0.000

**Table 3:** Comparison of stone clearance rate and complications between the two groups.

Group	Number	Stone clearance rate (number/%)	Blood transfusion rate (number/%)	Interventional embolization rate (number/%)
Experimental group	63	57/90.48	1/1.59	0/0.00
Control group	55	44/80.00	3/5.45	2/3.64
*P* value	0.106	0.517	0.215	0.106

## Discussion

Puncture along the axis of the targeted renal calyx is based on the same principle used to establish a percutaneous renal passage during percutaneous nephrolithotomy, which is derived from the anatomical structure of the kidney and blood vessels^3^. Cone-like pyramidal structures are found in the medulla of the kidney. The bottom of the pyramid is toward the cortex, and the tip of the pyramid forms the papilla. which inserts into the bottom of the conical calyx. The renal segmental artery and interlobar artery are shaped around the renal calyx and renal vertebrae. The axis passing through the targeted renal calyx also passes through the axis of the adjacent renal pyramidal body, which is the optimal anatomical route with less vascular distribution^7^. Meanwhile, the maximum angle and operating range of nephroscope can be reached throughout the puncture route in the axis of the renal calyx^7^.

In clinical practice, ensuring that the percutaneous renal puncture is routed precisely through the axis of the targeted renal calyx is difficult^8^. It requires an accurate location of the central axis of the 3D conical renal calyx and precise, simultaneous navigation in three dimensions (X, Y, Z). At present, the commonly used 2D guidance methods (i.e., plain radiography, B-US) cannot accurately locate the skin puncture point or route^9–11^, which is the most significant technical obstruction to using percutaneous renal puncture.

The radiography-guided puncture route and the axis of the targeted renal calyx are always in the same longitudinal plane, although these two lines cannot be guaranteed to overlap in this plane. It often takes multiple puncture points, multiple angles, and repeated attempts to penetrate the targeted renal calyx, and it often deviates from the axis of the targeted renal calyx, resulting in injury and bleeding of the blood vessels around the renal vertebrae and renal calyx, which limits the scope of nephroscopy, increases the difficulty of puncture, and affects the efficiency of stone removal.

Although US guidance can show the targeted renal calyx at multiple angles and monitor the puncture needle through the center line of the 2D section of the targeted renal calyx^8^, it is difficult to determine whether the 2D section selected by US contains the axis of the targeted renal calyx, which is also the geometric axial section of the pyramidal targeted renal calyx. Especially for a targeted renal calyx with hydronephrosis, which deforms into an oval body or sphere, it is even more difficult to locate the axial section of the targeted renal calyx using US. Thus, it could be concluded that, with US guidance, it is often impossible to pass the axis of the targeted renal calyx accurately, resulting in injury to, and bleeding from, the blood vessels around the renal vertebrae and renal calyx, as well as allowing only limited intraoperative nephroscope movement.

Accurate target percutaneous renal calyx longitudinal puncture has always been the biggest challenge in percutaneous renal surgery. In recent years, a variety of percutaneous renal puncture navigation systems have emerged, including electromagnetic positioning navigation^12^ and iPad augmented reality navigation^6^ and so on. The above techniques can achieve fast and safe puncture in the operation to some extent, but their disadvantages are also significant. iPad augmented reality auxiliary system needs complex equipment, software and professional personnel. In addition, due to the movement of renal position caused by respiratory activity during the operation, there is a certain deviation between the augmented reality image and the actual position of the patient, while the iPad augmented reality auxiliary system does not have real-time position tracking of the puncture needle, which may cause the puncture needle to deviate from the target puncture line. Electromagnetic positioning and navigation technology also requires complex electromagnetic equipment and professionals, and the flexible ureteroscope needs to be placed into the target renal calyx in advance. Ureteral stricture and kidney stone obstruction will limit its use. In addition, because there is no real-time monitoring, it is difficult to ensure that the adjacent organs of kidney are not damaged during the puncture. The above shortcomings limit the clinical application of these techniques, resulting in their exploratory application in only a small number of patients.

The fusion of virtual 3D images and real-time ultrasound images may be the development direction of percutaneous renal puncture navigation in future. Based on the above assumption, we designed 3D visualization technology combined with real-time ultrasonic monitoring to navigate percutaneous renal puncture more precisely via three standardized steps for the first time. First, we calculated and marked the geometric axis of the targeted renal calyx and then marked the optimal puncture point on the skin using the grid surface patch. Second, we measured and segmented the optimal simulated guided section image and matched the tangent precisely on the skin using the grid location patch. This tangent line is the optimal placement position of the US probe on skin. Third, we moved the US probe around the puncture point and along the localization tangent. By fusing and comparing the observed sections of the collecting system with the optimal simulated guiding cut section segmented using 3D visualization technology, we determined the optimal direction of the US probe. Throughout these steps, the navigational information gained by 3D visualization technology is fused with real-time guidance of B-US, thereby successfully taking advantage of the rapid, stereoscopic, accurate percutaneous renal puncture navigation.

In this study, because we accurately combined the positioning function of 3D visual image with the real-time ultrasonic monitoring function, we overcame the respective shortcomings of 3D image and ultrasonic localization, and realize the accurate location of skin puncture point and the accurate guidance of puncture path. First of all, the postoperative puncture channel is highly in line with the longitudinal axis of the target renal calyx, which meets the requirements of accurate percutaneous renal puncture. Secondly, on the basis of accurate puncture, compared with the standard B-US-guided puncture, the loss of hemoglobin in the experimental group decreased significantly, suggesting that the 3D visualization fused with ultrasound can reduce the risk of bleeding. Thirdly, he primary channel success rate in the experimental group was significantly improved, and the puncture time and operation time were significantly reduced compared with the control group, which indicated that the 3D visualization fused with ultrasound could reduce the technical difficulty of percutaneous renal puncture, provided a set of standardized operation process of percutaneous renal puncture, and was conducive to more surgeons to master percutaneous renal puncture. In addition, although there was no difference in the stone clearance rate, the blood transfusion rate, interventional embolize rate between the experimental group and the control group in statistics, the powers of these were very low, indicating that this result may be due to the extremely low incidence, limited sample size and other factors.

It is easy to apply and popularize the 3D visualization fused with ultrasound. Although seem a bit complex, the steps of the 3D visualization fused with ultrasound take only 30 min for each patient. Only computer, 3D software, and simple learning for software operation are needed.

## Conclusions

In summary, 3D visualization fused with ultrasound can help overcome the limitations of current conventional 2D guidance methods, as it preliminarily realized accurate navigation of puncture through the axis of the targeted renal calyx. The clinical application effect was good preliminarily, and its further clinical use will verify its accuracy.
